# Malignant Pediatric Gliosarcoma Defies General Survival Data

**DOI:** 10.1155/2014/175679

**Published:** 2014-12-17

**Authors:** Jovita Martin, Premkumar Devadoss, Kalaichelvi Kannan, Suresh Kumar Sundarraj

**Affiliations:** ^1^Department of Medical Oncology, Madras Medical College, Chennai, India; ^2^Madras Medical College, Chennai, India

## Abstract

Gliosarcoma, a variant of glioblastoma multiforme, is a dimorphic tumor known for its intra-axial occurrence and poor survival of less than a year. Here is an 11-year-old boy with gliosarcoma. He had a near total excision and postoperative chemoradiotherapy. He has lived through the disease for over 34 months with a residual disease.This case report is to report an unusual long survival of gliosarcoma in a teenager (Ravisankar et al., 2012).

## 1. Introduction

Gliosarcoma is known for its rarity occurrence in older individuals and poor survival. It constitutes only 2% of aggressive astrocytomas or glioblastoma [1–4]. Even the best of multidisciplinary approaches have resulted in bleak survival rates. This pediatric case report is reported for its rarity of occurrence and survival rate in spite of residual tumor.

## 2. Case Report

**Figure 1:**
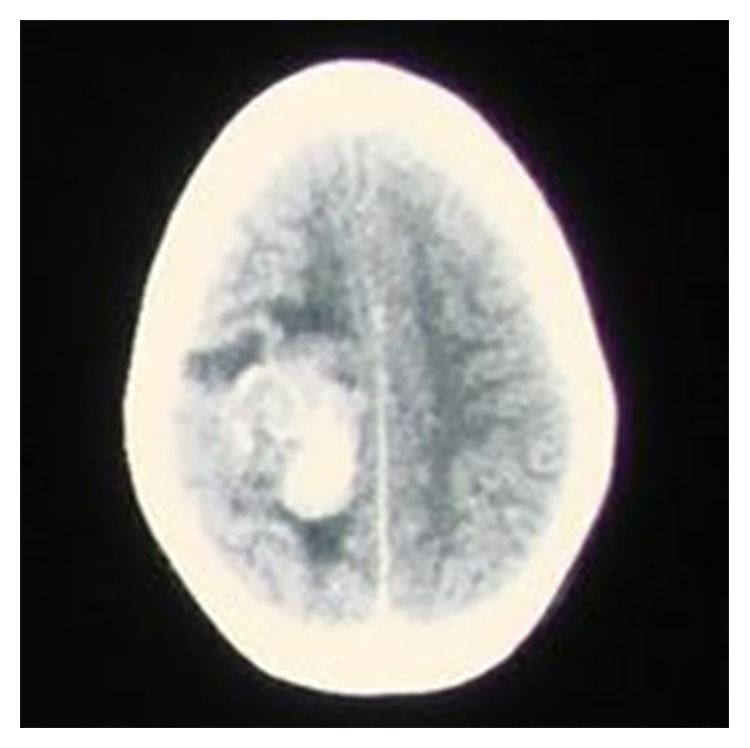
Preoperative CT scan brain showing a calcified lesion in the right temporoparietal region.

**Figure 2:**
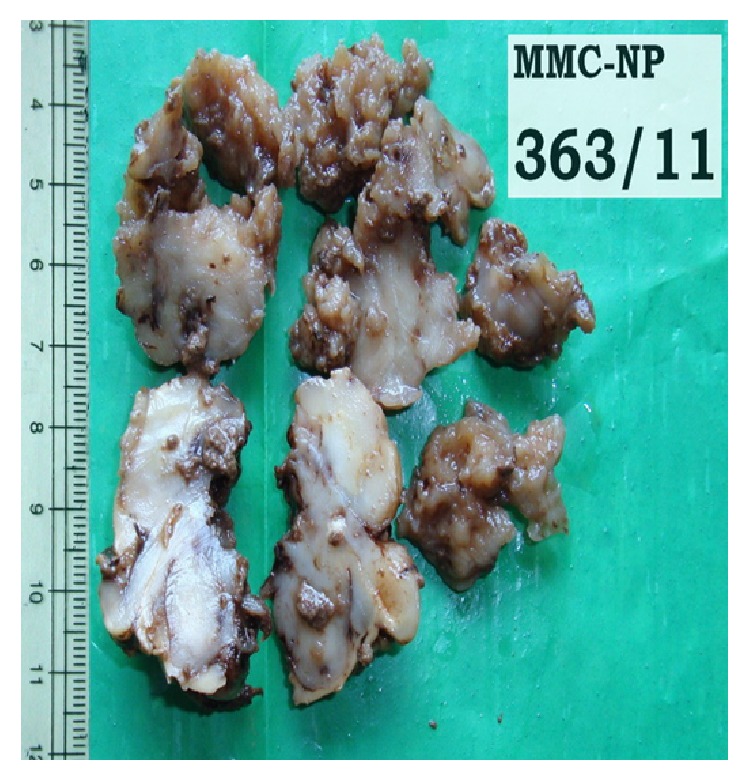
Gross post-op specimen.

An 11-year-old boy presented to the outpatient department with headache and vomiting in August 2011 [1]. CT scan showed a contrast enhancing isodense space occupying lesion with areas of calcification in right frontal cortex with surrounding edema (Figure 1). Craniotomy was done and part of the lesion was sent for squash cytology. The smear appeared cellular showing discohesive sheets and clusters of pleomorphic oval to polygonal cells with abundant eosinophilic cytoplasm some showing cytoplasmic vacuolation and marked nuclear atypia in a background of necrosis and hemorrhage. A suggestion of atypical teratoid/rhabdoid tumour was made. A near total excision of the space occupying lesion in the right parietooccipital region was done and sent for histopathologic examination. Grossly, the specimen was received as multiple irregular gray white soft tissue fragments ranging from 0.5 × 0.5 cm to 4 × 3 × 2 cm. External surface appears irregular and nodular. Cut surface appears variegated with gray tan areas, glistening areas, cystic areas, and hemorrhagic areas. Few areas of calcification are also seen (Figure 2).

**Figure 3:**
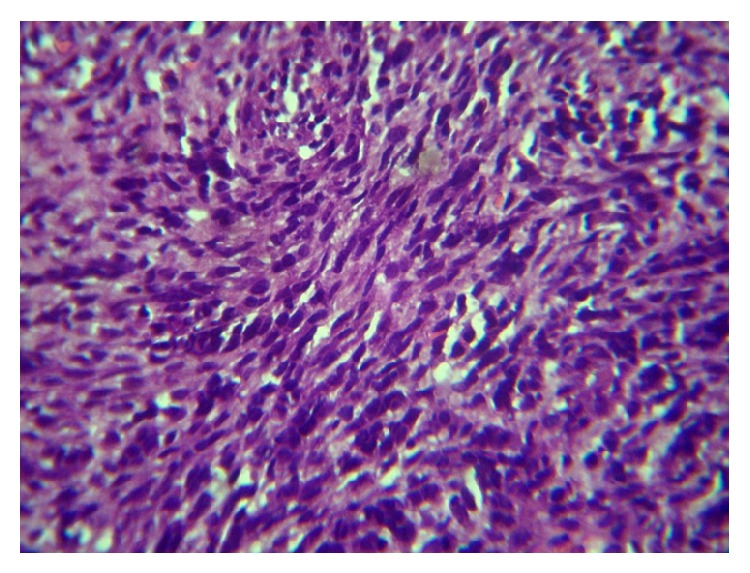
10x with mitosis.

Hematoxylin and eosin stained tissue sections revealed a highly cellular neoplasm composed of predominantly spindle shaped cells with pleomorphic oval to elongated hyperchromatic nuclei (Figure 3). The cells are arranged in interdigitating fascicles and herring bone pattern in few foci and with frequent mitoses of 2–10 per high power field. There are also foci showing deep staining round cells arranged in small clusters and rosettes with areas of necrosis and hemorrhage and no reticulin fibers present.

A differential diagnostic consideration of (1) gliosarcoma with leiomyosarcomatous differentiation and (2) teratoma with malignant transformation was made.

**Figure 4:**
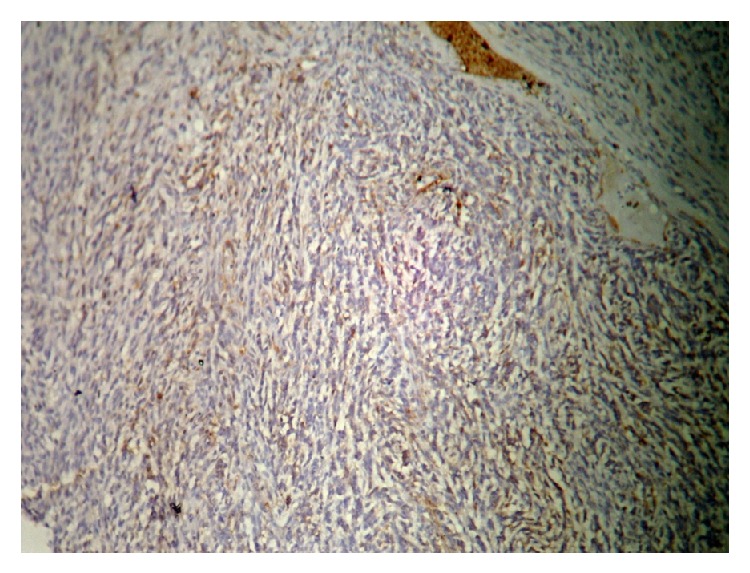
SMA.

**Figure 5:**
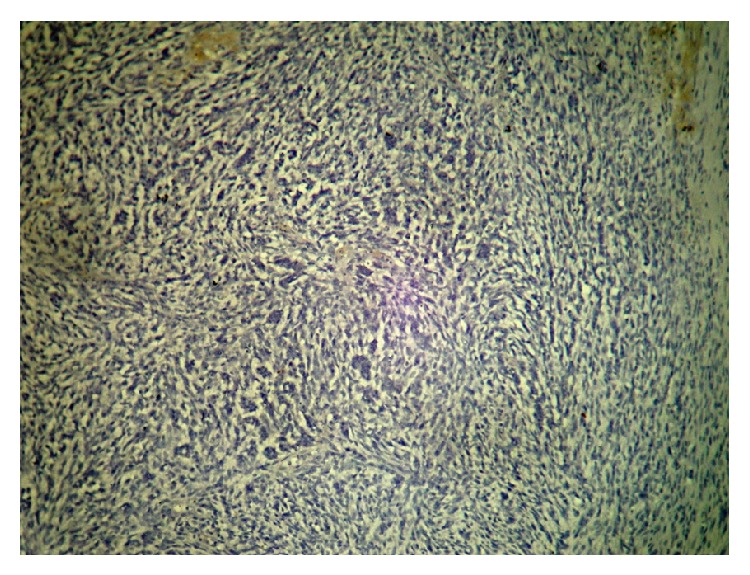
GFAP.

**Figure 6:**
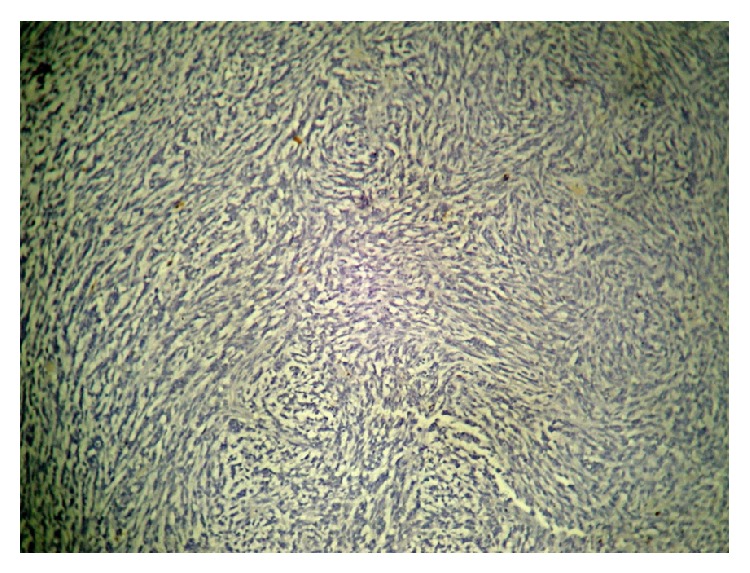
Mgen.

**Figure 7:**
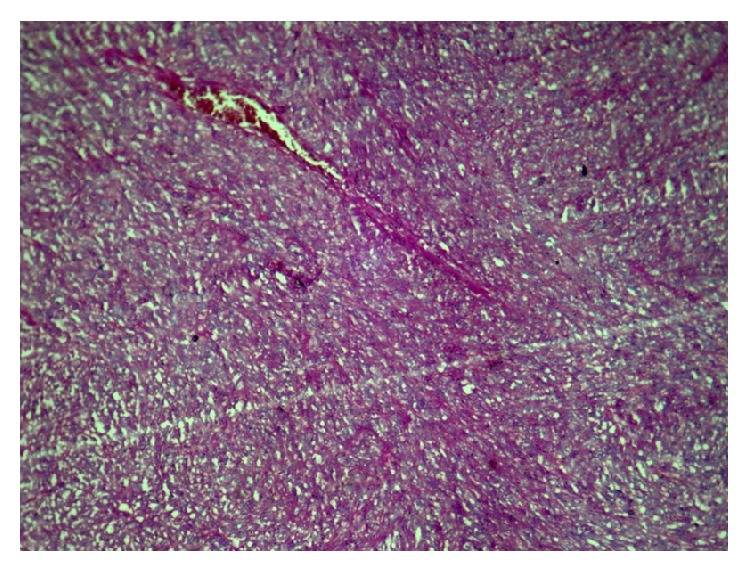
PAS.

**Figure 8:**
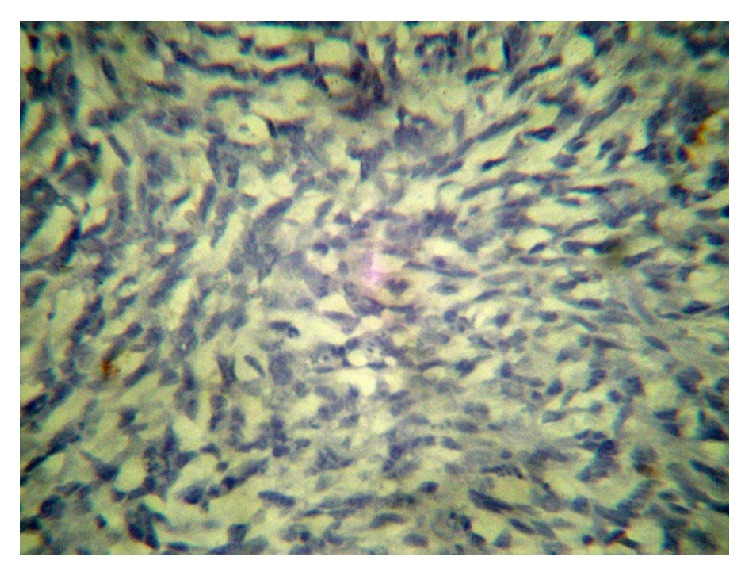
S100.

**Figure 9:**
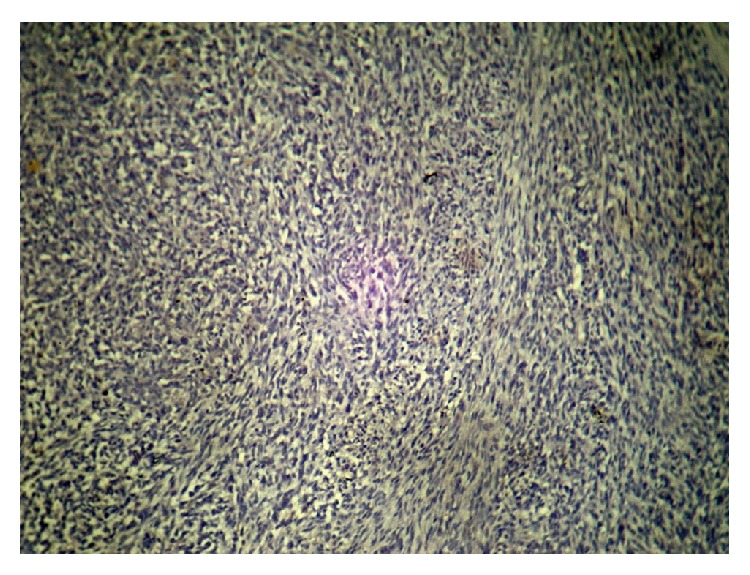
EMA.

Immunostaining for Vimentin showed cytoplasmic positivity in 70% of the cells. Smooth muscle actin showed focal weak positivity in 30% of the cells. Immunostaining for S100, epithelial membrane antigen, was found to be negative and glial fibrillary acidic protein was scanty positive; Ki67 proliferation index was 10–15% and P 53 protein was positive (Figures 4, 5, 6, 7, 8, and 9). A final diagnosis of gliosarcoma was then made.

**Figure 10:**
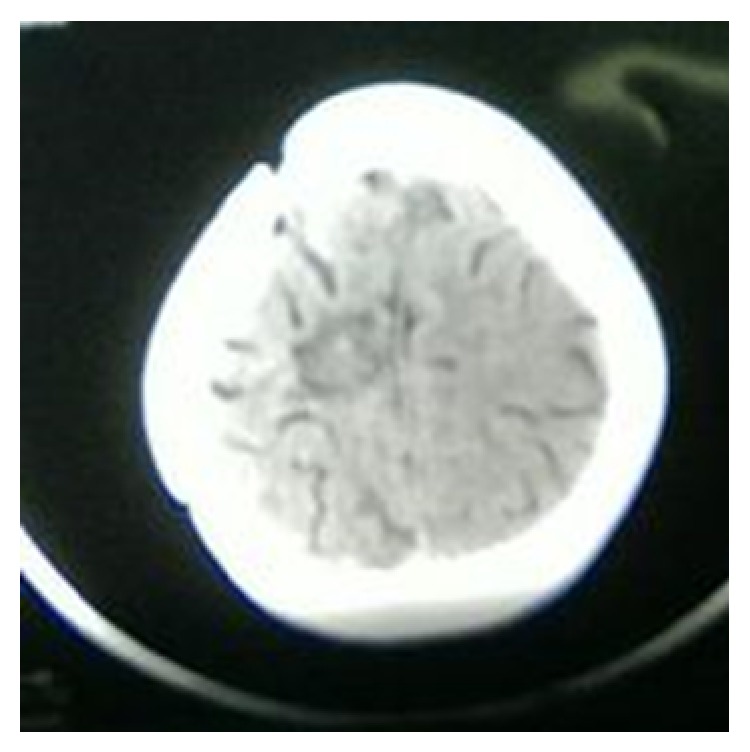
Post-op CT scan brain with residual disease.

**Figure 11:**
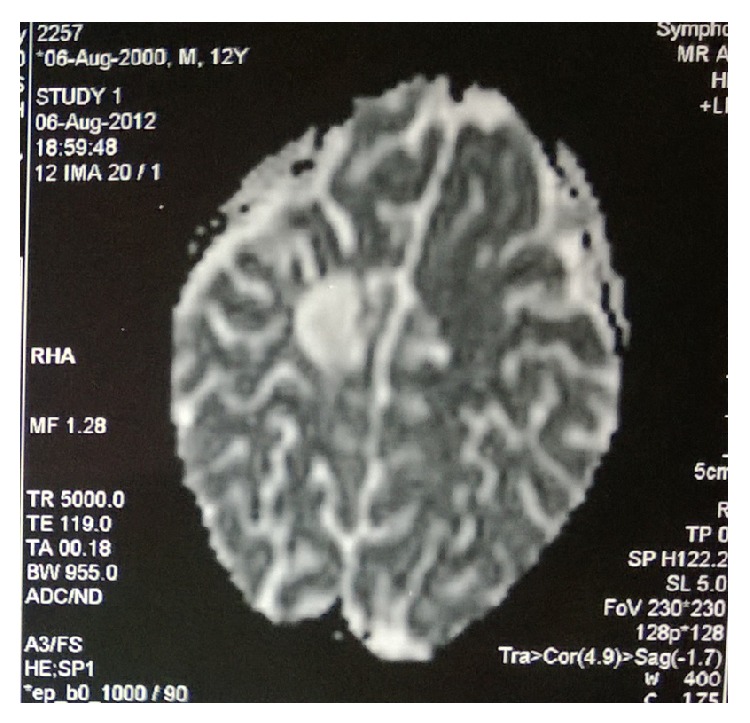
CT scan residual tumor 2012.

**Figure 12:**
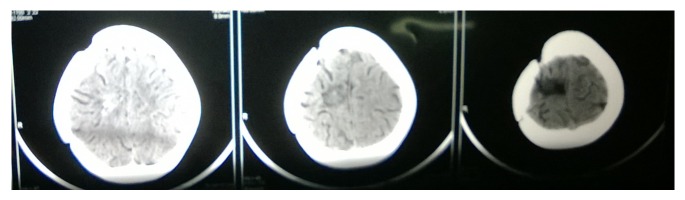
CT scan brain with residual tumor in the right temporoparietal region May 2014.

The patient was given external beam radiotherapy 66 Gy at 2 Gy in 33 fractions and 6 cycles of chemotherapy with temozolomide 200 mg per day for 5 days every 28 days (Figure 10). The post-treatment MRI scan showed a residual focal area of brain edema and granulomatous calcified lesion with reactivation 2.5 × 2.5 cm; the lesion is hypointense in T1W and hyper in T2W (Figure 10). On examination his performance score according to Eastern Cooperative Oncology group is 2, he has a scar on the right side scalp consistent with the previous surgery, his cognition is good, and he has mild difficulty in talking and mild weakness in the left lower limb. He is on regular follow-up with us and has survived for 30 months (Figures 11 and 12).

## 3. Discussion

Gliosarcoma or Feigin tumor, a variant of glioblastoma multiforme, constitutes 5–10% of gliomas [4]. Gliosarcoma is by itself a rare tumor, which occurs with a male predilection with a median age of 52.1 years with a mean survival of 8.3 months [5, 6]. Pediatric gliosarcomas are very rare; however few cases of congenital gliosarcomas are reported and 3 cases of long term survivors of more than 5 years are reported after complete resection [6–10]. This tumor is known to occur preferentially in temporal lobe and causes dissemination and extracranial metastasis [11, 12].

The pathognomonic biphasic pattern of gliosarcoma constitutes the presence of gliomatous and sarcomatous components. There is also a theory of independently arising gliomatous and sarcomatous tumors called “collision tumor.” There is significant diagnostic dilemma which this entity poses on account of the gliomatous and mesenchymal components. The biphasic pattern is confirmed by reticulin fibers that highlight the reticulin-rich sarcoma component and glial fibrillary acidic protein (GFAP) positivity in immunohistochemistry, which demonstrates the glial component. Gliosarcomas also present with adenoid formation signifying metastatic carcinoma, with chondroid elements such as osseous metaplasia and with smooth muscle elements such as rhabdomyosarcomatous [13–15].

The treatment for gliosarcoma is through multimodality approach. Complete resection followed by chemoradiation is a must. However most of the patients portend dismal prognosis [16, 17]. Literature review by Isaacs elucidates the overall survival as 28% [18, 19].

## 4. Conclusion

Pediatric gliosarcoma is evidently rare, and survival dictated by the disease is poor even with the best of multimodality approaches. This case of an 11 years old boy in his preadolescence treated with near total excision and chemoradiotherapy which has weathered the storm of the disease surviving to live his adolescence with good performance despite his residual disease is worth reporting.
